# Negative and Positive Selection Pressure During Sexual Transmission of Transmitted Founder HIV-1

**DOI:** 10.3389/fimmu.2019.01599

**Published:** 2019-07-10

**Authors:** Bernadien M. Nijmeijer, Teunis B. H. Geijtenbeek

**Affiliations:** Department of Experimental Immunology, Amsterdam University Medical Centers, Amsterdam Infection and Immunity Institute, University of Amsterdam, Amsterdam, Netherlands

**Keywords:** dendritic cell, langerhans cell, transmitted founder HIV-1, IFITM, Type I IFN, Trim5a, viral restriction and dissemination

## Abstract

Sexual transmission of HIV-1 consists of processes that exert either positive or negative selection pressure on the virus. The sum of these selection pressures lead to the transmission of only one specific HIV-1 strain, termed the transmitted founder virus. Different dendritic cell subsets are abundantly present at mucosal sites and, interestingly, these DC subsets exert opposite pressure on viral selection during sexual transmission. In this review we describe receptors and cellular compartments in DCs that are involved in HIV-1 communication leading to either viral restriction by the host or further dissemination to establish a long-lived reservoir. We discuss the current understanding of host antiretroviral restriction factors against HIV-1 and specifically against the HIV-1 transmitted founder virus. We will also discuss potential clinical implications for exploiting these intrinsic restriction factors in developing novel therapeutic targets. A better understanding of these processes might help in developing strategies against HIV-1 infections by targeting dendritic cells.

## Introduction

**Figure 1:**
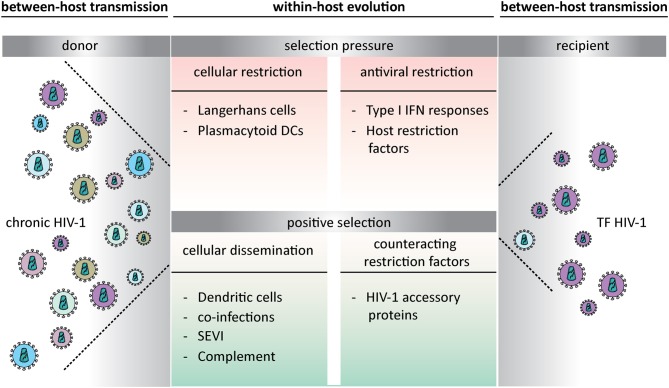
Opposing selection pressures during transmitted founder HIV-1 transmission at mucosal surfaces. The within-host evolution creates a selection bias that leads to either viral restriction or establishes a new infection. Transmitted founder viruses are able to escape host restriction and are therefore responsible for establishing productive infection. TF HIV-1, transmitted founder HIV-1; DCs, dendritic cells; IFN, Interferon; SEVI, semen-derived enhancer of virus infectivity.

The number of new HIV-1 infections globally continues to decline. From a peak of 3.4 million new infection a year in 1996 to 1.8 million in 2017. The intervention of early combination antiretroviral therapy (cART) is clinically beneficial to patients and very effective in preventing HIV-1 transmission (1–3). The introduction of pre-exposure prophylaxis (PrEP) will further interfere with HIV-1 transmission (4–6). However, currently there is no curative treatment or vaccine to prevent HIV-1 infection. Uncovering the mechanisms underlying viral transmission and pathogenesis is crucial to develop methods to prevent HIV-1 transmission. Sexual transmission of HIV-1 results most commonly from virus exposure at mucosal surfaces (7, 8). The identification of transmitted founder (TF) viruses emphasizes the existence of selection pressure mechanisms that lead to the transmission of only specific HIV-1 strains (9). Host factors influence whether virus exposure leads to productive infection. These may include the physical barrier of the mucosa (10), the amount of available target cells (11), altered mucosal microbiota (12, 13), and immune activation by genital inflammation established by other sexual transmitted infection (14–18). Also, genital fluids are known to contain proteins that enhance viral infection, like semen-derived enhancer of virus infectivity (SEVI) and complement (Figure 1) (19, 20). The transmission risk is associated with the specific within-host barriers, which creates a selection bias with an advantage for viruses with higher between-host transmission potential (21–24). Important cells that exert opposing selection pressures are the different dendritic cell (DC) subsets localized in the mucosal tissues.

## Virus-Host Interactions at Mucosal Sites

DC subsets play an important role in transmission of viruses such as HIV-1 across mucosal tissues (14, 25). The cellular plasma membrane is the first interaction of HIV-1 with its host and an important step in viral transmission and pathogenesis. HIV-1 spreads either as cell-free virus particles or via cell-cell transmission. While cell-free virus allows spread of virions in more distant tissues, cell-cell transmission is much more efficient and it helps the virus to overcome physical and immunological barriers (26). *In vitro* studies illustrate that cART and broadly neutralizing antibodies (bnAb) do not neutralize HIV-1 cell-cell transmission as potent as cell-free spread (27–29), which underscores the importance of understanding the mode of viral transmission for drug design.

The compartments where fusion of HIV-1 with the host cell occurs differs and is dependent on the cell type and mode of viral transmission. For CD4 T cells, HIV-1 fuses at the plasma membrane using the CD4 receptor and a co-receptor such as CCR5 and CXCR4 (30–32). For DCs viral fusion occurs at the plasma membrane (33) or after internalization via clathrin mediated endocytosis (34, 35). Internalization minimizes the exposure of viral epitopes at the cell surface, thereby reducing the efficacy of inhibitors targeting these epitopes (35). In contrast, endocytosis does not always lead to productive infection. When HIV-1 is endocytosed in multivesicular bodies (MVBs) the virus can be recycled back toward the plasma membrane for transfer to uninfected cells (36, 37). However, internalization can also lead to autophagic destruction in specific cells (38). Indeed, different DC subsets have distinct roles in HIV-1 dissemination because of the differences in handling the virus. Mucosal langerhans cells (LCs) capture and internalize HIV-1 leading to degradation, thereby preventing HIV-1 dissemination (38, 39), whereas DCs play a key role in transmitting the virus to target CD4 T cells.

## Dendritic Cells Facilitate HIV-1 Dissemination upon Sexual Contact

DCs patrol the submucosal tissues to capture invading pathogens for antigen presentation to T cells in the lymph nodes, thereby facilitating HIV-1 transmission (Figure 1) (40). DCs facilitate viral transmission to T cells either by HIV-1 fusion and productive infection of the DC, leading to viral transmission to permissive cells or by capture and internalization of HIV-1 into MVBs and transmission independent of DC infection (36, 37, 41). Besides their role in HIV-1 dissemination they are also important in triggering an innate immune response upon viral exposure. DCs express relatively low levels of the CCR5 and CXCR4 co-receptor and CD4 entry receptor, which could account for the lower levels of infection seen in DCs (42). DCs express many attachment molecules that mediate virus internalization and transfer. Indeed, the C-type lectin receptor (CLR) DC-specific intercellular adhesion molecular 3-grabbing non-integrin (DC-SIGN) is thought to play an important role in HIV-1 binding and internalization via endocytosis into clathrin coated pits (41, 43, 44). After internalization the virions can stay infectious for many days and can be transmitted to CD4-positive T cells (45). In this way DCs serve as virus reservoirs to mediate *trans*-infection of CD4-positive T cells, thereby facilitating spread of HIV-1 to the lymph nodes (45, 46). DC-SIGN is highly expressed on *in vitro*-generated monocyte-derived DCs (moDCs), at mucosal sites on CD14-positve dermal DCs (dDCs) and on sub epithelial-based vaginal myeloid DCs (47–49). For DCs that do not express DC-SIGN (50), different attachment receptors like Siglec-1 (CD169) have been identified to transfer HIV-1 (51, 52). Also external factors can promote *trans*-infection, like semen fluid, which contains fragments of prostatic acid phosphatase forming amyloid aggregates termed SEVI that promote viral attachment to DCs thereby increasing *trans*-infection of CD4-positive T cells by HIV-1 (19, 53). There are different processes described by which *trans*-infection occurs. One process is internalization via clathrin-mediated endocytosis (41, 43, 44). Antigen bound to DC-SIGN in mature DCs localizes in compartments with a neutral pH close to the cell surface, which could represent early endosomes (43). In contrast, in immature DCs DC-SIGN co-localizes with late endosomes or lysosomes (41). A different *trans*-infection route is dependent on invagination. For mature DCs, HIV-1 is internalized in a CD81 tetraspanin rich compartment, which is distinct from endocytic vesicles but adjacent to the plasma membrane (36, 54). This allows protected transfer of virions by DCs and delivery to target cells in the lymph node. Interestingly, more recently it has been shown that a process called micropinocytosis is involved in transfer of HIV-1 from immature DCs to CD4-positive T cells. Dynamin 2 (DNM2), a gene involved in organelle and membrane trafficking limits HIV-1 endocytosis and maintains virions on the surface of DCs for efficient transfer (55). Once in the lymph node HIV-1 can be transmitted from DCs to T cells via different mechanisms. DC-SIGN-bound HIV-1 facilitates optimal contact with CD4 and CXCR4/CCR5 co-receptors on T cells, enhancing viral transfer (56), HIV-1 is transferred via virological synapses which is formed by direct contact between DCs and T cells (57–59) or DCs transfer HIV-1 to T cells via exosomes (60, 61). Interestingly, exosomes derived from HIV-1 infected cells contain HIV-1 viral genome (62) and are able to establish productive infection in target cells (61, 63). All these mechanisms contribute to viral escape and promote further dissemination in the newly infected host.

## Langerhans Cells are Either Protective Against or Promote HIV-1 Transmission

LCs a subset of DCs are abundantly present at epithelia of vagina, foreskin and within the anal tissues (64, 65) and under normal conditions are therefore the first immune cells to encounter HIV-1 during sexual transmission (38, 39). Langerin (CD207) is a CLR expressed almost exclusively by LCs and is important for antigen capture and internalization, which induces Birbeck granules formation and routing of antigen into organelles (66). In contrast to DC-SIGN, langerin has a role in antiviral protection as immature LCs do not become infected by HIV-1 but capture HIV-1 via langerin, leading to TRIM5α-mediated degradation of HIV-1 and thereby preventing HIV-1 dissemination (Figure 1) (38, 39). LCs from inner foreskin explant cultures and vaginal explant are not productively infected by HIV-1 but several studies suggest that these cells support *trans*-infection of CD4-positive T cells (64, 67). Since the restrictive nature of LCs is dependent on the activation state and can be saturated, the amount of virus and isolation method could explain differences observed in restriction and infection (68). Taken together, the outcome of these studies suggest that immature LCs generally seem to be more restrictive to HIV-1 infection, whereas activation of LCs allows *cis*-infection and subsequent transmission of HIV-1 to T cells (14, 64). Inflammatory stimuli like TNFα, Pam3CSK4 or Interleukin-7 increase HIV-1 transmission by increasing HIV-1 replication or capture (14, 69). Also, viral coinfections, such as HSV-2, breach the protective function of LCs by abrogating langerin function, which increases HIV-1 susceptibility (15, 65). This implies that activation of LCs by inflammation or genital co-infection alters the protective function of LCs, mediating HIV-1 transmission (Figure 1), which might be associated with lower expression of langerin on activated LCs as langerin has anti-viral properties (39).

## Interferon Pressure at Mucosal Sites

Viral infections sensed by pattern recognition receptors (PRRs) lead to the activation of signaling cascades that results in the release of interferons (IFNs). Upregulation of type I IFN production is one of the earliest innate responses observed in HIV-1 infection. Production of type I IFNs during viral infections promotes an antiviral environment by an autocrine feedback loop triggering the IFN receptor and subsequently inducing cellular expression of IFN-stimulated genes (ISGs) within the infected cells but also in bystander cells (70). Several studies have shown that HIV-1 is able to escape intrinsic IFN-β response triggering by limiting replication of viral DNA (71) or actively blocking virus sensing by PRRs, which contributes to efficient HIV-1 replication (72). Besides cellular IFN responses upon viral infection, plasmacytoid dendritic cells (pDCs) secrete a second wave of type I IFNs in response to viruses or tissue damage (73, 74). pDCs develop in the bone marrow and circulate in the blood. Macaque studies have shown that upon SIV exposure, pDCs are recruited to the mucosal sites of virus transmission, become activated and start producing high levels of type I IFNs (75). The outcome of this high IFN response by pDCs has conflicting functions in antiviral defense. Some of the induced ISGs act as host restriction factors to prevent HIV-1 infection and dissemination. In contrast, during acute infections, IFN produced by pDCs results in maturation of bystander myeloid DCs that play a crucial role in transporting the virus to secondary lymphoid organs thereby promote transmission (Figure 1) (74). It has been shown that DCs upregulate the interferon-inducible receptor Siglec-1 which is able to transfer HIV-1 to T cells (76). Also, studies show that ISGs are upregulated during chronic infection (77, 78). The persistent activation of pDCs during chronic infection may contribute to immune activation and inflammation, which is associated with AIDS disease progression (79, 80). These consequences of high IFN production promote viral dissemination. Earlier studies suggested that IFN-α responses in mucosa of non-human primates could enhance infection and the IFN-α induction did not protect animals from SIV infection (75, 81). However, IFN production may also create an antiviral environment. Recently an elegant study showed that early type I IFN responses in macaques prevent SIV infection and slow disease progression (82). Moreover, in uninfected but high exposed individuals, higher IFN-α levels have shown to be protective against infection (83). Also, the induction of an effective early antiviral immune response at mucosal sites creates selective pressure for viruses that are resistant to type I IFN (84).

## Transmitted Founder Viruses are Responsible for Initial HIV-1 Infection

In 60–80% of mucosal infection, a single specific HIV-1 variant, the TF virus, establishes productive clinical infection (Figure 1) (9, 85, 86). To be able to cross intact mucosal barriers TF viruses have specific properties that provide an advantage to establish new infections more efficiently (87, 88). TF virus strains are relatively resistant to IFN compared to viruses isolated later in infection (84, 88–90), suggesting adaptations in HIV-1 evolution to escape host restriction. TF viruses replicate and spread more efficient in CD4 T cells in the presence of IFN-α than chronic viruses (84, 88). This suggest that IFN resistance of TF viruses is specifically important during initial infection as type I IFNs are produced at lower levels during systemic infection when chronic viruses replicate. Also, initial HIV-1 infection occurs predominantly with R5 HIV-1 strains (31, 91) and TF viruses have the chemokine receptor 5 (CCR5) tropism (9, 92). TF viruses incorporate more envelope glycoprotein (Env) per particle compared to chronic HIV-1 viruses, which is associated with enhanced infection of target CD4 T cells (88). Furthermore, it has been shown that TF viruses bind more efficiently to DCs than their chronic counterparts giving TF viruses a potential selection advantage in transmission to a new host (88). Phenotypic analyses of TF viruses show an enhanced resistance to fusion inhibitors, masking of CCR5 co-receptor binding sites, and more neutralizing antibodies compared to chronic HIV-1 strains (9, 93). Since TF viruses need to establish infection they might have specific capabilities to infect immune cells such as DCs an LCs. Moreover, certain TF virus strains might infect immature LCs more efficient compared to their chronic counterparts, which could indicate that TF viruses might have an intrinsic capacity to escape LC restriction (Figure 1). These findings underscore the importance of LCs as initial targets for sexual transmission of HIV-1 and understanding these phenotypic properties of TF viruses is essential for vaccine design. Especially in the era of PrEP, transmitted drug resistance could be of concerns as it could select for higher virulent TF viruses (94).

## Host Antiretroviral Restriction Factors Against HIV-1

Host restriction factors play an important role in suppressing retroviral replication and dissemination (Figure 1). Many restriction factors that target HIV-1 are induced by type I IFN. Well-known HIV-1 restriction factors in DCs are IFITM (Interferon-induced transmembrane proteins), TRIM5α (E3-ubiquitin ligase tri-partite-containing motif 5a) (38) SAMHD1 (SAM- and HD domain-containing protein 1) (95), APOBEC3 (apolipoprotein B mRNA-editing enzyme, catalytic polypeptide-like 3) (96), Mx2 (Myxovirus resistance 2), and bone marrow stromal antigen 2 (BST2 or Tetherin) (97). Because of the potent antiviral potential of IFN many viruses have developed mechanisms to promote their survival. HIV-1 although sensitive to type I IFNs, is able to antagonize host restriction factors that inhibit virus entry to facilitate viral dissemination (Figure 1) (98). HIV-1 accessory proteins well known to counteract important restriction factors are: viral protein R (Vpr) viral infectivity factor (Vif) which antagonizes APOBEC3 proteins, negative regulatory factor (Nef) and viral protein unique (Vpu) antagonizing BST2 (99, 100).

IFITMs are small membrane-associated cellular factors that inhibit the replication of HIV-1 at the entry step (101). IFITMs do not block the internalization of viruses but inhibit fusion of the virus with the host cell. Whether HIV-1 is sensitive to IFITM restriction is determined by the subcellular localization of the IFITMs and HIV-1 co-receptor usage (102). TF viruses are more resistant to the antiviral activity of IFITMs. The ability by TF viruses to evade IFITM restriction is due to its relative resistance to IFN. Interestingly, IFITM restriction contributes to the increased IFN sensitivity of chronic HIV-1 viruses (102).

TRIM5α targets incoming retroviral capsid before integration to block infection. TRIM5α expression levels and polymorphisms have been associated with the clinical course of HIV-1 infection in cohort studies underscoring the antiviral effect of TRIM5α (103–105). Unique about TRIM5α is that it can restrict diverse retroviruses in a species-specific manner. Rhesus TRIM5α (rhTRIM5α) strongly restricts HIV-1, whereas human TRIM5α has been thought to have poor restriction activity against HIV-1 (106). More recently some primary isolates of HIV-1 have been found to be more sensitive to human TRIM5α restriction than lab strains (107, 108). So restriction of TRIM5α on replication may vary according to the virus. The functional capacity of TRIM5α also depends on the localization of the restriction factor in the cell. It has been suggested that non-human primate DCs lack efficient TRIM5α mediated retroviral restriction because TRIM5α is unable to restrict incoming viruses because it is absent from the cytoplasm (109). TRIM5α localization to the nucleus triggers induction of type I IFN during infection (109). Notably, recent data show that TRIM5α restriction might be cell specific. Immature LCs protect against HIV-1 infection by inducing langerin-mediated autophagic degradation of captured HIV-1 (38). The LC specific restriction factor TRIM5α is dependent on the CLR function. HIV-1 binding to Langerin routes HIV-1 into the TRIM5α mediated restriction pathway which targets virions for degradation and thereby prevents infection of LCs. Taken together, the outcome of these studies support a role for human TRIM5α in HIV-1 transmission and pathogenesis *in vivo*.

SAMHD1 is highly expressed in myeloid cells like DCs and macrophages (95). SAMHD1 also targets the early phase of viral infection as it inhibits reverse transcription by depleting the pool of cellular dNTPs (95, 110, 111). HIV-2 viral protein X (Vpx) is able to counteract SAMHD1 restriction. Degrading SAMHD1 by treating DCs with SIV-Vpx leads to infection and maturation of DCs promoting viral dissemination (95). Whether HIV-1 infection leads to DC maturation is unclear as it has been shown that interfering with SAMHD1 restriction increases infection of DCs but not DC maturation (112). Furthermore, higher infection observed with SAMHD1 depletion correlates with a stronger suppression of maturation, suggesting that HIV-1 might actively suppress PRR sensing (112). HIV-1 complement opsonization bypasses SAMHD1 restriction in DCs by enhancing SAMHD1 phosphorylation, which results in DC infection (113).

Upon HIV-1 infection APOBEC3 is encapsulated into budding virions. In newly infected cells during reverse transcription of the viral RNA, APOBEC3G triggers G-to-A hypermutations leading to the production of defective proteins and non-functional virus particles which results in a strong inhibition of HIV-1 replication (96). Interestingly, exosomes can transfer host restriction factors such as APOBEC3 from cell to cell and thereby inhibit HIV-1 infection (114). Vif antagonizes APOBEC3 proteins by inducing the recruitment of proteins leading to polyubiquitylation and proteasomal degradation of APOBEC3, thereby preventing incorporation of APOBEC3 into virions (115, 116).

BST2 or Tetherin prevents the release mature Env virions by anchoring virions to the plasma membrane of infected cells (117, 118). The retention of viral particles at the plasma membrane leads to endocytic uptake and the accumulation of these virions in endosomes which may result in viral degradation and thereby inhibit the spread of newly formed virions (119). Similarly, Vpu interacts with tetherin, preventing tetherin trafficking to the cell surface, promoting ubiquitination and subsequent targeting to late endosomes and degradation in lysosomes (118, 120). This prevents incorporation of tetherin into virions thereby enhancing viral budding and release.

Accessory proteins positively contribute to transmission by allowing HIV-1 to escape host restriction. The continuous adaptation of HIV-1 to the antiviral activity of host restriction factors emphasizes their importance in controlling HIV-1 infection and viral transmission.

## Understanding Host-Virus Interactions for Specific Interventions

Mucosal DCs are among the first immune cells to encounter HIV-1 upon sexual contact. Therefore, receptors expressed or host antiviral factors induced by DCs or LCs could be used in immunotherapeutic strategies to prevent HIV-1 transmission. Langerin binds to glycan ligands for pathogen capture and internalization. A recent study identified chemical compounds with a high binding affinity to langerin (121). Interestingly, these compounds were found to modulate cellular signaling and to suppress inflammation (121, 122). Also, it has been shown that rhTRIM5α is very potent in HIV-1 restriction. Interestingly, human TRIM5α restriction is specific for LCs and is dependent on HIV-1 binding to langerin. Therefore, targeting langerin, host restriction factors like TRIM5α and other ISGs that contribute significantly to viral control could be interesting candidates for therapeutic applications (125). A better understanding of the specific properties of TF viruses, which will relate to different selection biases during transmission, will allow us to identify the specific selection mechanisms and thereby providing novel strategies to counteract the transmission of these TFs (24). The majority of TF viruses are of R5 tropisms and use CCR5 co-receptor for their initial infection, which makes CCR5 an interesting candidate for blocking early transmission. The higher incorporation of Env per particle may increase the sensitivity to neutralization by antibodies.

## Concluding Remarks

At mucosal sites DC subsets patrol the microenvironment and are therefore the first cells to interact with HIV-1 after exposure. If the virus carries specific properties and interacts with DCs or LCs determines the fate of the virus which can result in either routing of the virus for degradation or further dissemination. Strategies to counteract suppression mechanisms by HIV-1 leading to HIV-1 sensing and induction of type I IFN responses upon viral infection can be a powerful strategy to restrict viral dissemination. The induction of host factors and the ability of HIV-1 to counteract viral restriction shows the intricate interplay between HIV-1 and host. Further understanding of the specific within-host barriers provides new insights important for developing novel therapeutic approaches at the site of initial infection. Understanding the specific properties of TF viruses that create advantages to promote between-host transmission may contribute to the development of immunotherapeutic strategies to combat HIV-1 dissemination.

## Author Contributions

BN: designed and wrote the manuscript. TG: designed and edited the manuscript.

### Conflict of Interest Statement

The authors declare that the research was conducted in the absence of any commercial or financial relationships that could be construed as a potential conflict of interest.
